# Synchronous ipsilateral carcinoma of the accessory mammary gland and primary lymphoma of the breast with subsequent rectal carcinoma: report of a case

**DOI:** 10.1186/1477-7819-12-286

**Published:** 2014-09-14

**Authors:** Akihiro Nishikawa, Hide Kasai, Yoshinori Koyama, Naohiko Koide, Akihiro Iijima, Hisashi Shimojo, Shigeyoshi Kumeda

**Affiliations:** Department of Surgery, Nagano Prefectural Kiso Hospital, 6613-4 Fukushima, Kiso 397-8555 Japan; Department of Gastroenterology, Nagano Prefectural Kiso Hospital, 6613-4 Fukushima, Kiso 397-8555 Japan; Department of Pathology, Shinshu University School of Medicine, 3-1-1, Asahi, Matsumoto, 390-8621 Japan

**Keywords:** Synchronous malignancy, Accessory mammary gland, Breast carcinoma, Primary breast lymphoma, Rectal carcinoma, Diffuse large B-cell lymphoma, Invasive lobular carcinoma

## Abstract

A case of synchronous carcinoma of the accessory mammary gland and primary breast lymphoma with subsequent rectal carcinoma has not been reported previously. We present a very rare case of primary non-Hodgkin lymphoma of the left breast diagnosed simultaneously with invasive lobular carcinoma of the left axillary accessory mammary gland and rectal adenocarcinoma. An 82-year-old Japanese woman presented with two palpable masses on the left chest wall. She was given a diagnosis of suspected breast malignant tumor and axillary accessory mammary gland. She underwent excision of the axillary accessory mammary gland and left mastectomy with axillary lymph node dissection. Histopathological examination revealed diffuse large B-cell lymphoma of the breast and invasive lobular carcinoma of the axillary accessory mammary gland with lymph nodes metastasis. Three months after the surgery, primary rectal adenocarcinoma was also detected by F-18 fluorodeoxyglucose positron emission tomography. Hartmann’s operation was performed, since which time the patient has been doing well.

## Background

The synchronous occurrence of multiple neoplastic processes is uncommon, and coexistence with cancer and lymphoproliferative diseases of the breast is also unusual [1]. Furthermore, a carcinoma arising in the accessory mammary gland is rare, especially of the invasive lobular type [2]. We present an extremely rare case of synchronous primary non-Hodgkin lymphoma (NHL) of the left breast with invasive lobular carcinoma of the ipsilateral axillary accessory mammary gland, with subsequent rectal adenocarcinoma.

## Case presentation

An 82-year-old Japanese woman was referred to our hospital with two left breast masses. She had no previous breast problems or a family history of breast cancer. She had a history of persistent hepatitis C virus (HCV) infection, Alzheimer-type dementia, and left femoral neck fracture. No previous fever, night sweats, or weight loss was reported.

Physical examination revealed two masses on the left chest wall. One was a 3 × 3 cm, firm, freely mobile, and indolent mass in the upper outer quadrant of the left breast. The other was a 2 × 1 cm, elastic, and freely mobile mass in the lower part of the left axilla. The axillary mass was completely separate from the breast. Laboratory studies showed an increased soluble interleukin-2 receptor level of 547 U/ml, carcinoembryonic antigen of 5.4 ng/ml, and antibody to HCV (anti-HCV) with signal-to-cut-off ratio of 12.77. Mammography demonstrated a round, high-density, circumscribed mass, without microcalcification and spicula. Ultrasonographic examination of the breast tumor revealed an oval, hypoechoic, heterogeneous lesion, without posterior acoustic phenomena. Since fine-needle aspiration cytology of the breast tumor revealed that it could be categorized as being suspected of malignancy, left mastectomy with axillary lymph node dissection and excision of the axillary accessory mammary gland were performed.

**Figure 1:**
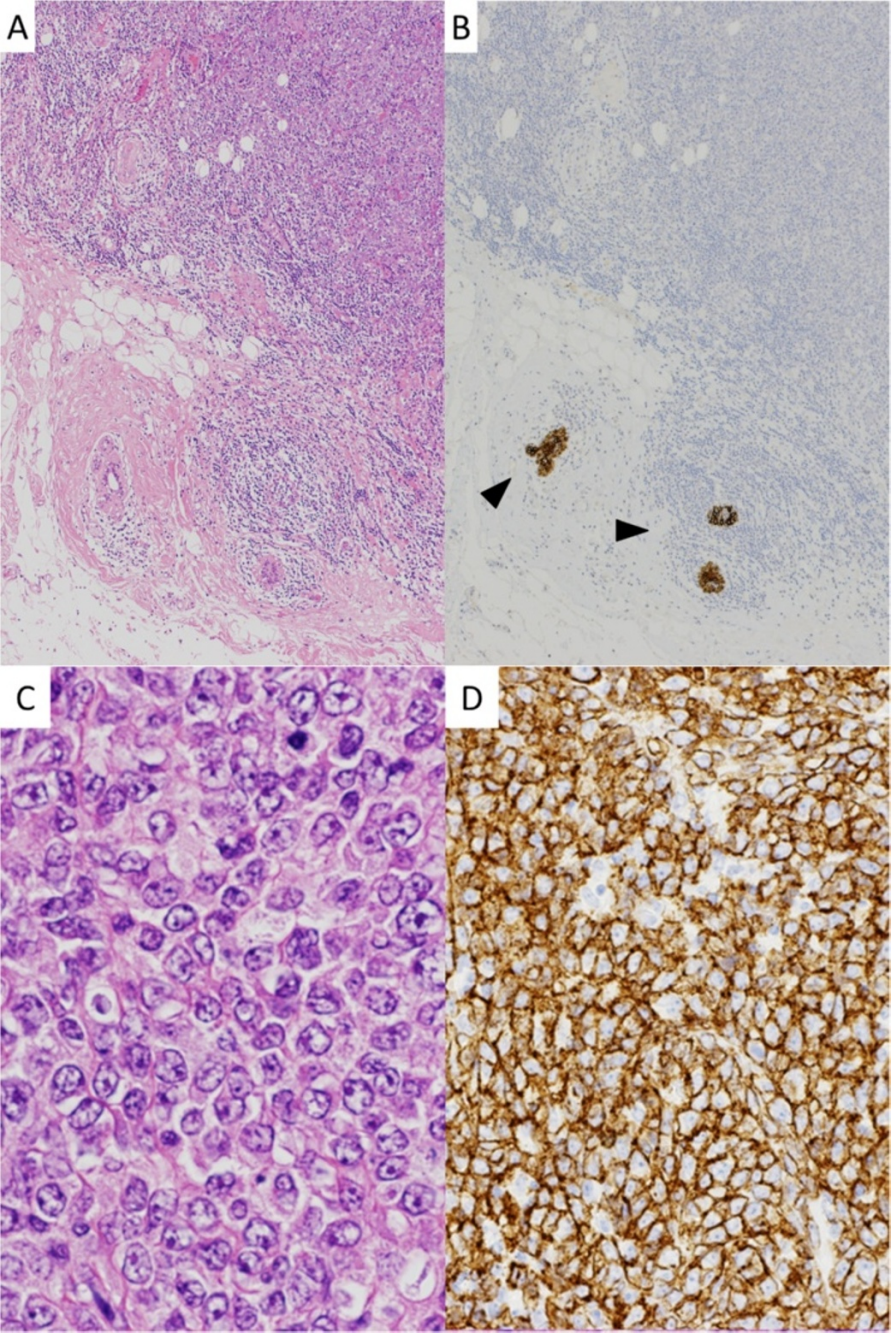
**Microscopic examination of the breast tumor. (A)** Atypical lymphoid cells have infiltrated diffusely into the mammary glands (hematoxylin and eosin stain, original magnification × 4). **(B)** Cytokeratin stain enhances mammary ducts (arrowheads) (cytokeratin AE1/AE3 immunostain, original magnification × 4). **(C)** The neoplastic cells have a large nucleus containing prominent nucleoli and vesicular chromatin (hematoxylin and eosin stain, original magnification × 40). **(D)** The cells are positive for CD20 (CD20 immunostain, original magnification × 20).

**Figure 2:**
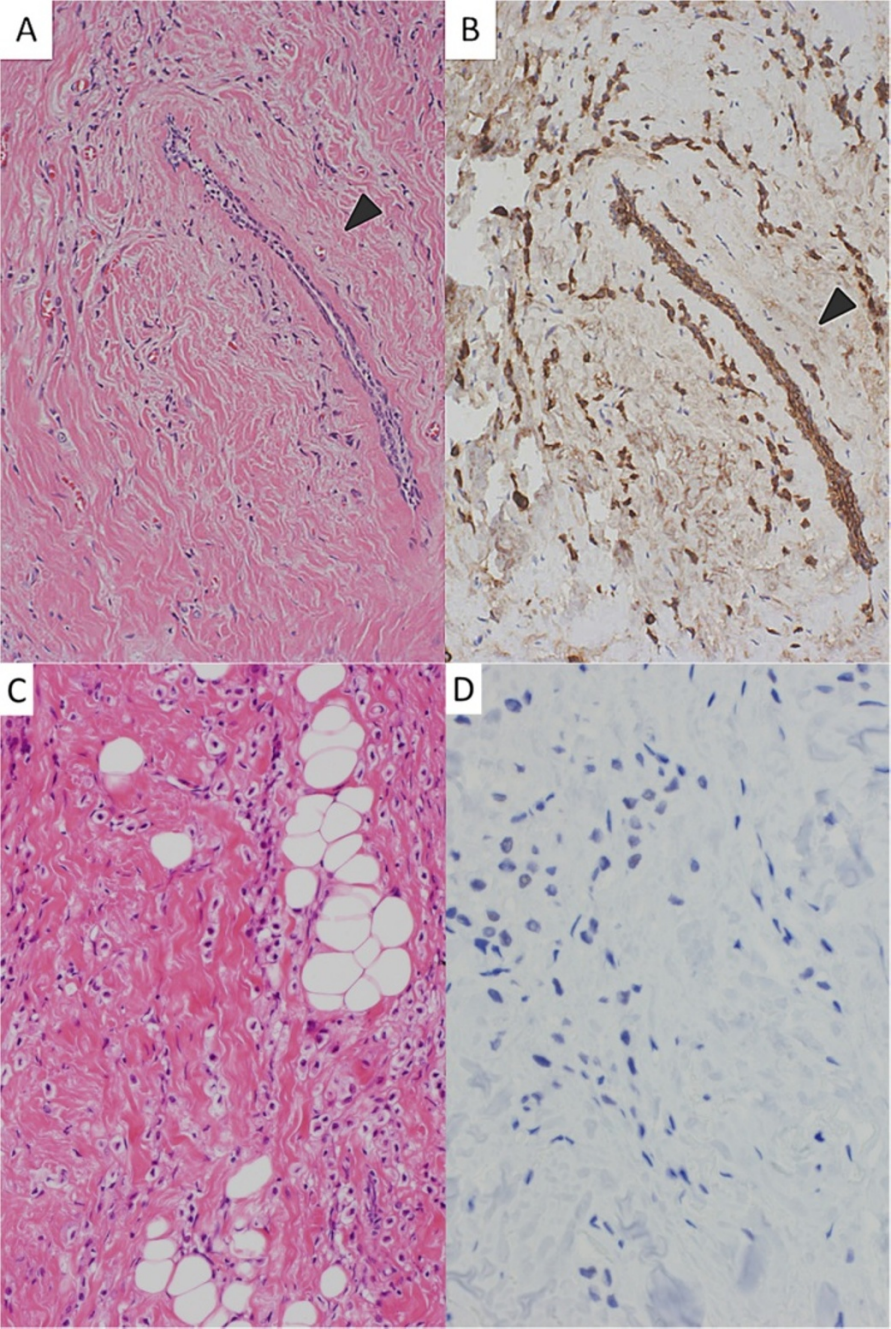
**Microscopic examination of the accessory mammary gland. (A)** The cancer cells have infiltrated around a mammary duct (arrowhead) (hematoxylin and eosin stain, original magnification × 10). **(B)** Cytokeratin stain enhances the cancer cells and the mammary duct. The mammary duct is adjacent to the cancer cells (arrowhead) (cytokeratin AE1/AE3 immunostain, original magnification × 10). **(C)** The cancer cells have pale to slightly eosinophilic cytoplasm and a nucleus with pale chromatin (hematoxylin and eosin stain, original magnification × 20). **(D)** The cancer cells are negative for E-cadherin (E-cadherin immunostain, original magnification × 20).

Gross examination of the breast mass revealed a white, firm tumor measuring 2.5 × 1.5 cm. Microscopic examination revealed diffuse sheet-like proliferation of atypical lymphocytes (Figure 1). The neoplastic cells were large with irregular nuclei containing prominent nucleoli and vesicular chromatin. Numerous mitotic figures were identified. On immunohistochemistry, the neoplastic cells were positive for CD20 and MUM1, and negative for CD3, CD5, CD10, and Bcl-6. These results confirmed the diagnosis of diffuse large B-cell lymphoma (DLBCL), not otherwise specified, and non-germinal center B-cell-like type. On the other hand, histopathology of the axillary tumor revealed ductal structures, fibrous tissue, fat tissue, and infiltrating cancer cells (Figure 2). The cancer cells showed dispersed or trabecular infiltrating growth in the fibrous tissue and the fat tissue. The cancer cells consisted of pale to slightly eosinophilic cytoplasm and a round nucleus with pale chromatin. On immunohistochemistry, the cancer cells were positive for cytokeratin AE1/AE3 and negative for E-cadherin. Furthermore, they exhibited a positive reaction to anti-estrogen receptor and anti-progesterone receptor, but were negative for HER2. These findings were consistent with invasive lobular carcinoma. All dissected lymph nodes were positive for metastatic lobular carcinoma.

**Figure 3:**
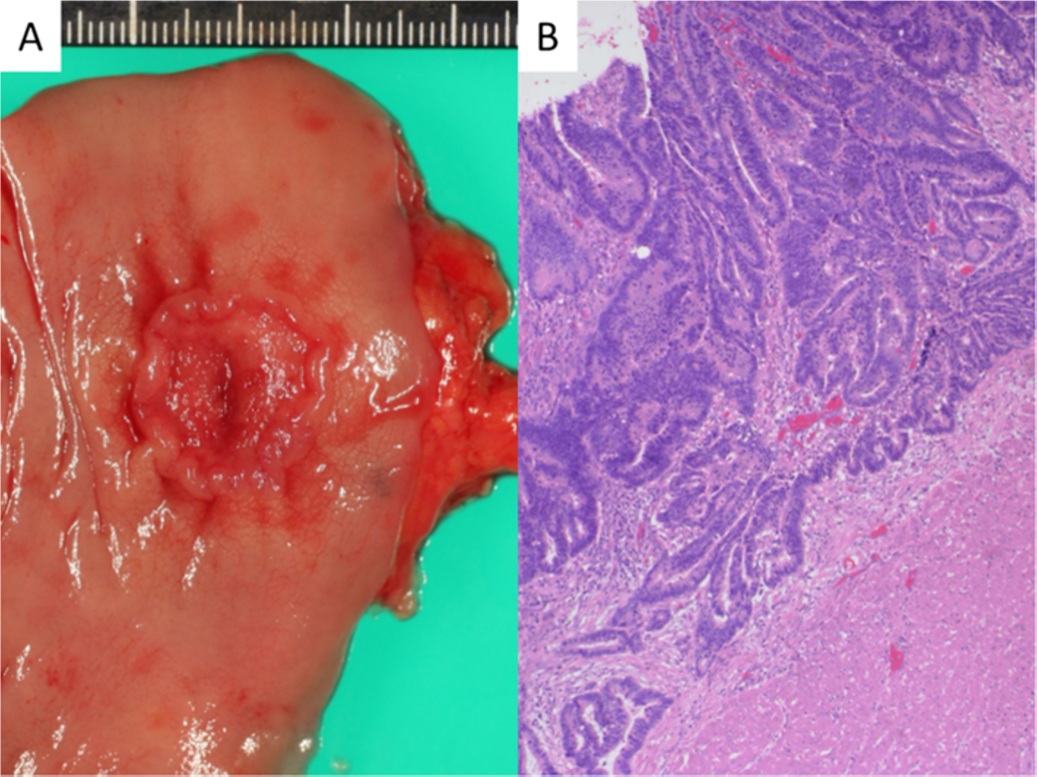
**Surgical specimen and microscopic examination of the rectal carcinoma. (A)** Surgical specimen showing that is a type 2 tumor measuring 15 × 15 mm. **(B)** Microscopic examination shows an adenocarcinoma forms moderate- to large-sized atypical glands. The carcinoma shows infiltrating growth in the muscularis propria (hematoxylin and eosin stain, original magnification × 4).

The patient underwent only adjuvant hormonal therapy with anastrozole at a dose of 0.79 mg/m^2^/day. F-18 fluorodeoxyglucose positron emission tomography 3 months after the surgery showed no evidence of distant metastasis of the carcinoma or infiltration of the lymphoma; however, it revealed focal accumulation in the lower rectum. Colonoscopy demonstrated a rectal carcinoma forming a fungating tumor with central ulceration. Hartmann’s operation was performed. The pathological findings revealed well- to moderately differentiated adenocarcinoma invading through the muscularis propria (Figure 3). There has been no recurrence or distant metastasis in the 7 months of follow-up since the last surgery.

## Discussion

Primary breast lymphoma (PBL) is a rare disease, accounting for 0.05% to 0.53% of all malignant breast tumors, 0.38% to 0.7% of all lymphomas, and 1.7% to 2.2% of all extranodal lymphomas [3–5]. The majority of cases are NHL and the most common histological subtype is DLBCL (40% to 70% of all PBLs) [6]. Extranodal marginal zone lymphoma of mucosa-associated lymphoid tissue (MALT lymphoma) is a distinct subgroup of PBL, accounting for 8.5% to 35% of all PBLs [6]. The diagnostic criteria for PBL proposed by Wiseman are the standard definition for this disease [7]. These criteria are: 1) presence of technically adequate pathologic specimens, 2) close association of mammary tissue and lymphomatous infiltrate, 3) no prior diagnosis of an extramammary lymphoma, and 4) no evidence of concurrent widespread disease, except for ipsilateral axillary lymph nodes if concomitant with the primary lesion.

The incidence of accessory mammary gland ranges from 0.6% to 6% of women in various ethnic groups [8]. Accessory mammary gland tissue is vulnerable to the same physiological and pathological changes, both benign and malignant, as normal breast tissue [9]. Cancer of the accessory mammary gland is a rare entity, accounting for 0.3% to 0.6% of all breast cancer [8]. The incidence of malignant change in the accessory mammary gland is unclear because the true incidence of accessory mammary gland is uncertain. The principal malignancy identified in accessory mammary gland is invasive ductal carcinoma (IDC) (79%), followed by medullary and lobular carcinomas, which are seen in less than 10% of cases [2]. Mitsuyoshi and colleagues advocated that cancer of the accessory mammary gland must be pathologically demonstrated to be adjacent to normal breast parenchyma that is not connected with normally positioned breast, and it is also necessary to rule out the possibility of a metastatic lesion from another primary cancer [10].

The synchronous occurrence of a carcinoma of the accessory mammary gland and PBL is an extremely rare finding, especially with a rectal carcinoma. To the best of our knowledge, this combination of synchronous double primary malignancies and rectal carcinoma has not been reported previously. We believe that this is the first reported case of its kind.

There have been only eight cases of the coincidence of breast cancer and PBL (excluding cases that did not satisfy Wiseman’s criteria for PBL, such as those with bone marrow infiltration); therefore, these are also rare entities [1, 11–17]. Herein, we summarize reported cases of synchronous double malignancies, along with our case (Table 1). The ages of the patients varied from 47 to 82 years of age, and the mean age was 61.9 years. Histology of PBL was B-cell NHL in all cases, and the major histological subtype was DLBCL (three cases). The majority of carcinomas were IDC, and only our case was invasive lobular carcinoma. Two neoplasms occurred ipsilaterally in seven cases, and in four cases the two tumors were close, within 2 cm of each other. In particular, in Case 4, the two tumors were invading each other and presenting as a “collision tumor”. Both Susnik and colleagues [18] and Anavekar and colleagues [19] reported cases of concurrent breast MALT lymphoma with bone marrow infiltration and ipsilateral breast cancer, respectively. Interestingly, the case of Susnik and colleagues exhibited a collision tumor like Case 4. In cases with the coincidence of primary or secondary breast lymphoma and breast cancer, MALT lymphoma may occur more frequently than expected.

**Table 1:** **Clinical characteristics of reported cases of synchronous primary breast lymphoma and breast carcinoma**

Case number [Reference]	Age/sex	Histology of PBL	Site of PBL	Histology of carcinoma	Size of PBL/carcinoma (mm)	Lymph node metastasis	Relationship between PBL and carcinoma	Remarks
1 [11]	49/F	Diffuse, mixed cell type, B-cell type	Left	IDC	50 × 45/unknown (not palpable)	Absent	Contralateral	-
2 [1]	62/F	B-cell NHL, diffuse high grade	Right	IDC	28/9	Absent	Contralateral	*
3 [12]	62/F	DLBCL	Right	IDC	29 × 28/13 × 10	Absent	Ipsilateral	-
4 [13]	53/F	MALT lymphoma	Left	IDC	25/25	PBL	Ipsilateral, colliding with each other	-
5 [14]	57/F	MALT lymphoma	Right	IDC + DCIS	Unknown (palpable)/8 right + 12 left	PBL	Ipsilateral, both in the same quadrant	-
6 [15]	47/F	B-cell NHL	Right	IDC	50 × 40 × 40/50 × 40 × 40	Absent	Ipsilateral, adjacent to each other	-
7 [16]	79/F	Large B-cell lymphoma of follicular cell origin	Bilateral	IDC	Both unknown (palpable)	Absent	Ipsilateral distance: 9 mm	†
8 [17]	66/F	DLBCL	Right	DCIS	20/unknown (palpable)	Absent	Ipsilateral distance: within 2 cm	-
Present case	82/F	DLBCL	Left	ILC	25 × 15/17 × 7	Carcinoma	Ipsilateral	‡

*Synchronous triple tumors, with bilateral Brenner tumors of the ovary. †Mouse mammary tumor virus sequences were identified. ‡The carcinoma occurred from the accessory mammary gland and posterior rectal cancer was diagnosed. DCIS, ductal carcinoma *in situ*; DLBCL, diffuse large B-cell lymphoma; F, female; IDC, invasive ductal carcinoma; ILC, invasive lobular carcinoma; MALT, mucosa-associated lymphoid tissue; NHL, non-Hodgkin lymphoma; PBL, primary breast lymphoma.

In Case 7, mouse mammary tumor virus (MMTV) sequences were detected by PCR of samples taken from IDC. MMTV has long been postulated as a causative agent of human breast cancer [20]. However, this remains controversial [20, 21]. Salmons and Gunzburg reviewed some recent reports in 2013 [21], and they did not accept the association with MMTV and human breast cancer, but suggested that it could be worthwhile to revisit these earlier studies. We did not attempt to detect MMTV sequences in our case.

There is also a possibility that HCV has been associated with B-lymphocyte proliferative disorders as well as various extrahepatic diseases [22]. On the strength of epidemiological data, emerging biological investigations, and clinical observations, Viswanatha and Dogan summarized and concluded that HCV was involved in the pathogenesis of at least a proportion of patients with NHL [23]. Okan and colleagues found that the anti-HCV seropositivity rate was significantly higher in the DLBCL subgroup (4 of 67 cases) than in the controls (9 of 802 cases) [24]. In our case, anti-HCV was examined four times in 1 year before and after admission, and all of the signal-to-cut-off ratios were about 10 and consistently high. This result suggests that the patient had had HCV infection before admission. There was a non-zero possibility that persistent HCV infection led to occurrence of the lymphoma.

Susnik and colleagues suggested that the coexistent carcinoma acted as an antigenic stimulant, which may have triggered the lymphomagenesis, a mechanism similar to the pathogenesis of acquired MALT lymphoma of the stomach as a result of the reaction to infection with *Helicobacter pylori*[18]. Since breast lymphoma usually grows rapidly [25], this suggestion is reasonable. By contrast, Broco and colleagues reported that ipsilateral IDC developed 1 year after an excision biopsy and conservative therapy of PBL, so prior lymphoma might also contribute to carcinogenesis [26]. From the above findings, both prior breast cancer and lymphoma may be possible causes of synchronous malignancy.

## Conclusion

We report an extremely rare case of synchronous ipsilateral carcinoma of the accessory mammary gland and PBL, with subsequent rectal carcinoma.

## Consent

Written informed consent was obtained from the patient for publication of this case report and any accompanying images. A copy of the written consent is available for review by the Editor-in-Chief of this journal.

## Abbreviations

anti-HCV: antibody to hepatitis C virus
DLBCL: diffuse large B-cell lymphoma
HCV: hepatitis C virus
IDC: invasive ductal carcinoma
MALT: mucosa-associated lymphoid tissue
MMTV: mouse mammary tumor virus
NHL: non-Hodgkin lymphoma
PBL: primary breast lymphoma
PCR: polymerase chain reaction.
